# The cGMP-Dependent Protein Kinase II Is an Inhibitory Modulator of the Hyperpolarization-Activated HCN2 Channel

**DOI:** 10.1371/journal.pone.0017078

**Published:** 2011-02-14

**Authors:** Verena Hammelmann, Xiangang Zong, Franz Hofmann, Stylianos Michalakis, Martin Biel

**Affiliations:** 1 Munich Center for Integrated Protein Science CIPSM and Department of Pharmacy – Center for Drug Research, Ludwig-Maximilians-Universität München, München, Germany; 2 Forschergruppe 923 Carvas, Technische Universität München, München, Germany; Dalhousie University, Canada

## Abstract

Opening of hyperpolarization-activated cyclic nucleotide-gated (HCN) channels is facilitated by direct binding of cyclic nucleotides to a cyclic nucleotide-binding domain (CNBD) in the C-terminus. Here, we show for the first time that in the HCN2 channel cGMP can also exert an inhibitory effect on gating via cGMP-dependent protein kinase II (cGKII)-mediated phosphorylation. Using coimmunoprecipitation and immunohistochemistry we demonstrate that cGKII and HCN2 interact and colocalize with each other upon heterologous expression as well as in native mouse brain. We identify the proximal C-terminus of HCN2 as binding region of cGKII and show that cGKII phosphorylates HCN2 at a specific serine residue (S641) in the C-terminal end of the CNBD. The cGKII shifts the voltage-dependence of HCN2 activation to 2–5 mV more negative voltages and, hence, counteracts the stimulatory effect of cGMP on gating. The inhibitory cGMP effect can be either abolished by mutation of the phosphorylation site in HCN2 or by impairing the catalytic domain of cGKII. By contrast, the inhibitory effect is preserved in a HCN2 mutant carrying a CNBD deficient for cGMP binding. Our data suggest that bidirectional regulation of HCN2 gating by cGMP contributes to cellular fine-tuning of HCN channel activity.

## Introduction

Hyperpolarization-activated cyclic nucleotide-gated channels (HCN1-4) comprise an ion channel family of four distinct members that pass a current termed I_h_ or I_f_ [1], [2], [3], [4]. I_h_ is widely found in nervous system and heart and has been known to play a key role in controlling cardiac and neuronal rhythmicity (“pacemaker current”) [4], [5]. Besides its pacemaker function, I_h_ contributes to other basic neuronal processes, including determination of resting membrane potential [6], [7], [8], dendritic integration [9], [10] and synaptic transmission [11]. Impaired function of HCN channels has been implicated in the pathologies of epilepsies, neuropathic pain disorders, and cardiac arrhythmia [2], [3].

Structurally, HCN channels belong to the 6 transmembrane ion channel superfamily. HCN channels are set apart from other members of this family by their unusual activation process that includes principal gating by membrane hyperpolarization (conferred by a transmembrane voltage sensor) and modulation of the voltage-dependence of activation by binding of cyclic nucleotides to the C-terminal cyclic nucleotide-binding domain (CNBD). The latter process is of crucial relevance because it connects HCN channel activation to numerous signal transduction pathways that control cellular levels of cAMP or cGMP.

There is recent evidence that HCN channel activity is also subject to regulation by protein kinases. For example, in hippocampal pyramidal neurons, the activation of p38 MAPK shifts the activation curve of I_h_ towards more positive potentials [12]. There are also some reports on protein kinase A-mediated phosphorylation of HCN channels [13], [14], [15]. Recently, the Src tyrosine kinase has been identified as another modulator of HCN channel gating [16].

Given these findings, we were wondering whether HCN channels may be regulated by additional, not yet specified proteins, and in particular by protein kinases. We focused our study on the HCN2 channel isoform because this channel is the most widely expressed HCN channel type in brain and heart [17], [18]. We provide evidence for the functional interaction between HCN2 and the cGMP-dependent protein kinase II (cGKII). Importantly, we demonstrate that cGKII-mediated phosphorylation of HCN2 shifts the voltage-dependence of channel activation to more negative voltages and, hence, counteracts the stimulatory action of cyclic nucleotides conferred by the CNBD. We propose that bidirectional regulation of HCN channel activation by cyclic nucleotides plays an important role in regulating the set point and threshold of HCN channel activation in neurons.

## Results

### The HCN2 channel interacts with cGKII via its proximal C-terminus

**Figure 1:**
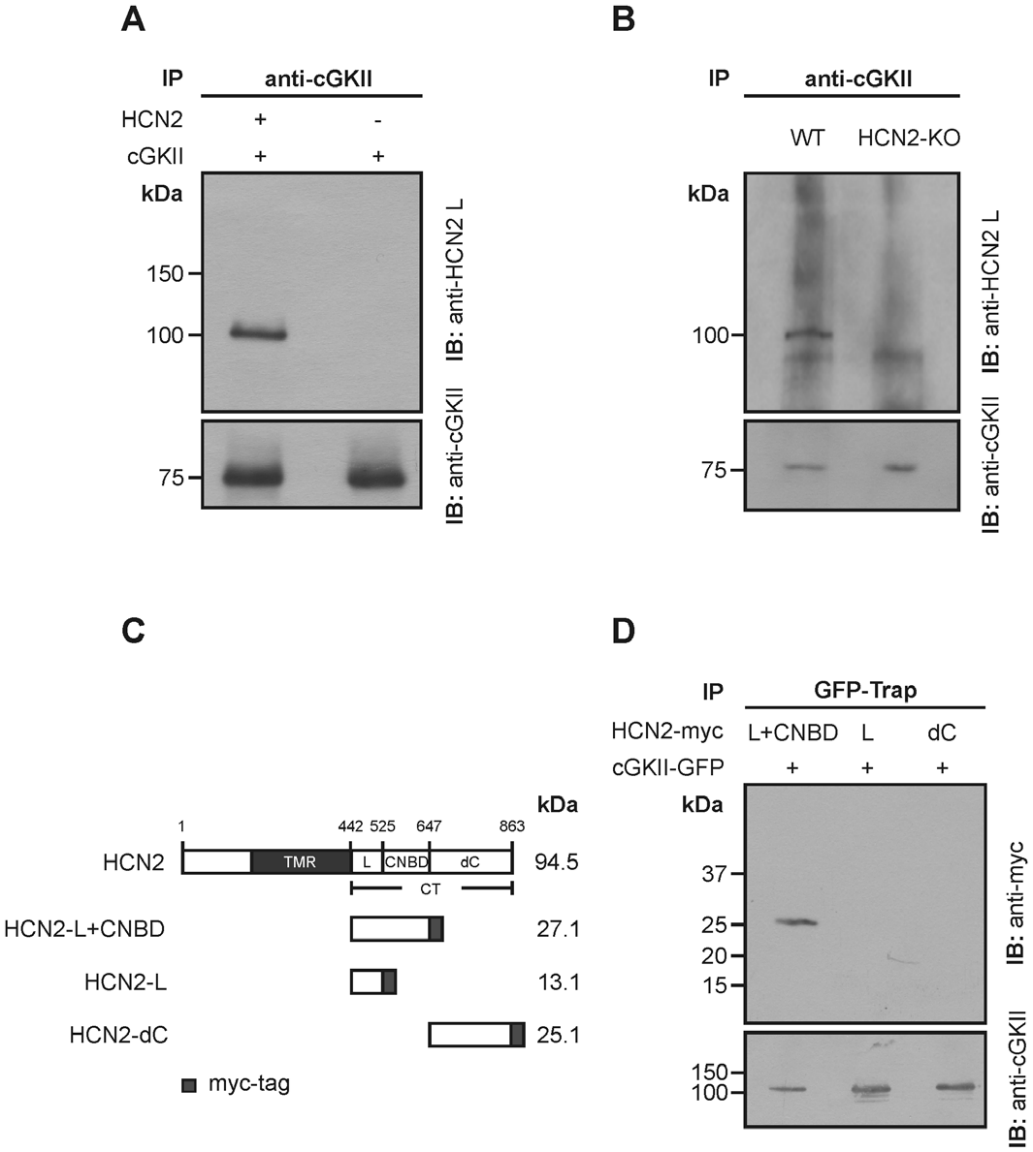
Interaction between HCN2 and cGKII. (**A**) Coimmunoprecipitation of HCN2 and cGKII in HEK293 cells. Lysates of HEK293 cells transfected with HCN2 and cGKII or cGKII alone were immunoprecipitated (IP) using a cGKII antibody and stained for HCN2 and cGKII as loading control. 500 µg protein was applied per lane. (**B**) Protein extracts of hypothalamic brain tissue from WT and HCN2-KO mice were immunoprecipitated using a cGKII antibody and analyzed in immunoblots (IB) for HCN2. Anti-cGKII served as loading control. (**C**) Schematic representation of full length HCN2 (862 amino acids) and myc-tagged HCN2-domains used for interaction studies. The calculated molecular size of the proteins is indicated. NT, N-terminus; TMR, transmembrane region; CT, complete HCN2 C-terminus; L, C-linker; CNBD, cyclic nucleotide-binding domain; dC, distal C-terminus. (**D**) GFP-Trap. Lysates of HEK293 cells coexpressing cGKII-GFP and myc-tagged portions of the HCN2 C-terminus were bound to GFP-tagged beads. Co-immunoprecipitated proteins were detected by immunoblotting with an anti-myc antibody. Anti-cGKII was used as loading control.

In a screen to identify protein kinases interacting with HCN channels, we coexpressed HCN2 and cGKII in HEK293 cells. Upon coimmunoprecipitation (Co-IP) with an anti-cGKII antibody, a 100 kDa band corresponding to HCN2 was detected in immunoblots (Fig. 1A). To verify a specific interaction of the two proteins we performed Co-IP experiments with anti-cGKII antibody in lysates from mouse hypothalamus, a brain region known to express both HCN2 and cGKII [19], [20]. Again, a specific HCN2 band was detected (Fig. 1B, left lane) confirming an *in vivo* interaction of HCN2 and cGKII. Importantly, the HCN2 band was not present in hypothalamic tissue from HCN2-deficient mice (Fig. 1B, right lane).

To further narrow down the region of HCN2 that interacts with cGKII, Co-IPs with GFP-tagged cGKII and myc-proteins corresponding to the combined C-linker/cyclic-nucleotide binding domain (L+CNBD, aa 443–647), the C-linker (L, aa 443–525) or the distal C-terminus of the HCN2 channel (dC, aa 647–863) were performed (Fig. 1C, D). Distinct bands were obtained for the combined C-linker/CNBD region (Fig. 1D, left lane) while no interaction was found for the C-linker alone (Fig. 1D, middle lane) as well as for the sequence downstream of the cyclic-nucleotide binding domain region (Fig. 1D, right lane). Together, these findings indicated that the CNBD is required for the interaction with cGKII, either alone or in conjunction with the C-linker. Attempts to perform Co-IPs with the isolated CNBD failed because of problems with protein stability (data not shown).

### HCN2 and cGKII are coexpressed in mouse brain

**Figure 2:**
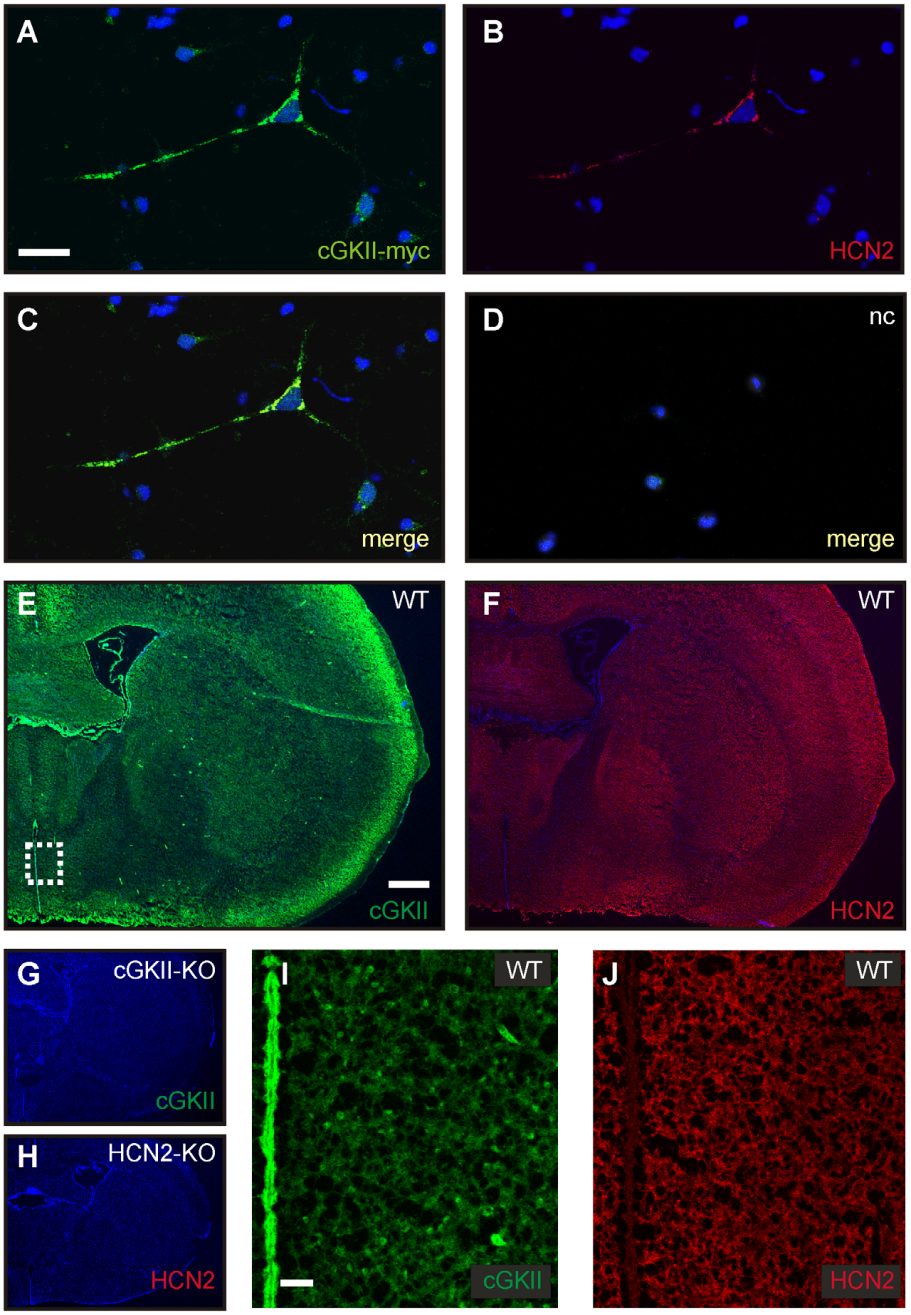
Colocalization of HCN2 and cGKII in neurons. (**A–D**) Colocalization in primary neurons. Hippocampal neurons of neonatal mice (E16.5) were cotransduced with lentivirus expressing HCN2 and cGKII-myc, respectively. Neurons were stained with antibodies against myc (**A**) and HCN2 (**B**). Counterstaining was performed with Hoechst dye. (**C**) Merge of (**A**) and (**B**). (**D**) Negative control (nc). Merge of stainings in the absence of primary antibodies. Scale bar corresponds to 100 µm. (**E–H**) Immunohistochemical staining of coronal brain slices. Consecutive slices from wild-type mice were stained with anti-cGKII (**E**) or anti-HCN2 (**F**). The signal was amplified by tyramide signal amplification. Counter stain was performed with Hoechst 33342 nuclear dye. As negative control, coronal slices of cGKII-KO (**G**) and HCN2-KO mice (**H**) were used. Scale bar corresponds to 500 µm. (**I, J**) Higher magnification of cGKII (**I**) and HCN2 (**J**) staining in the hypothalamic region corresponding to the dotted white box as indicated in (**E**). Scale bar corresponds to 50 µm.

In order to study the subcellular localization of cGKII and HCN2, primary hippocampal neurons were cotransduced with recombinant lentiviral particles expressing the HCN2 and a cGKII-myc fusion protein, respectively. Subsequent immunocytochemical staining showed colocalization of the two proteins at the plasma membrane (Fig. 2A–C). In the absence of primary antibodies immunostaining was not observed, demonstrating the specificity of the antibodies used (Fig. 2D). Immunohistochemistry revealed broad expression of cGKII (Fig. 2E) and HCN2 (Fig. 2F) in coronal sections of mouse brain. No specific staining was observed in brain sections of mice deficient for cGKII (Fig. 2G) or HCN2 (Fig. 2H), respectively. High levels of cGKII mRNA have been reported to exist in thalamic and hypothalamic regions [19]. In agreement with this finding, we observed coexpression of high levels of cGKII and HCN2 protein in consecutive slices covering the hypothalamus (Figs. 2I and 2J).

### HCN2 is phosphorylated by cGKII at position S641

**Figure 3:**
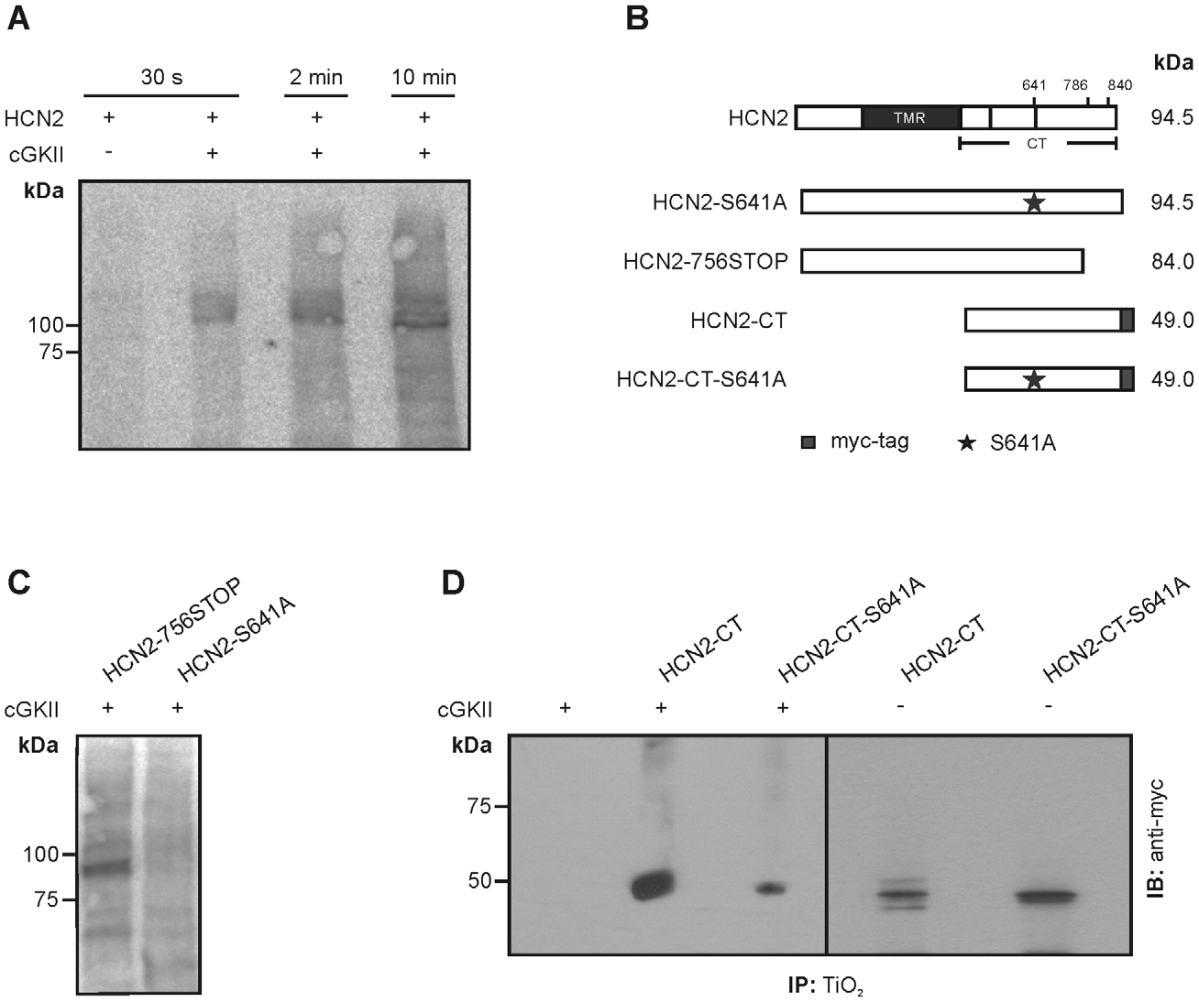
Phosphorylation of HCN2 by cGKII. (**A**) In vitro phosphorylation of HCN2 by cGKII. Lysates of COS-7 cells expressing HCN2 and cGKII were incubated with [γ-32P]-ATP for the times indicated. After incubation, proteins were separated on SDS page and analyzed by autoradiography. The first lane represents a control reaction with a cell lysate lacking cGKII. (**B**) HCN channel constructs used for phosphorylation studies. The positions of the three putative cGKII phosphorylation sites (S641, S786 and S840) are indicated. The calculated molecular mass is given for each construct. (**C**) Phosphorylation assay of a HCN2 mutant lacking S786 and S840 (first lane) and the HCN2-S641A mutant. (**D**) Pulldown of phosphoproteins by TiO_2_ beads. Lysates of cells expressing HCN2-CT or HCN2-CT-S641A in the presence or absence of cGKII, respectively, were incubated with TiO_2_ beads. Proteins specifically bound to the beads were analyzed with an anti-myc antibody.

We next tested whether HCN2 can be phosphorylated by cGKII. In lysates of HEK293 cells coexpressing HCN2 and cGKII a 100 kDa phosphorylated protein band corresponding to HCN2 appeared after the addition of [γ-^32^P]-ATP. By contrast, in lysates lacking cGKII the 100 kDa HCN2 band was not observed (Fig. 3A). HCN2 contains three serines that are located within a consensus site (K/R-K/R-X-S/T) for phosphorylation by cGKs (S641, S786 and S840; Fig. 3B). Serine 641 is located at the distal end of the α-C helix of the CNBD and is present in all four members of the HCN channel family. By contrast, the two distal consensus sites (S786 and S840) are not conserved throughout the HCN channel family (HCN1 and HCN3 contain no phosphorylation consensus sites at positions equivalent to S786 or S840; HCN4 contains only the consensus site at the position equivalent to S786). A HCN2 truncation mutant lacking the last two serines (HCN2-756STOP) was still efficiently phosphorylated by cGKII (Fig. 3C, left lane). By contrast, a cGKII-dependent phosphorylation of HCN2 was not detectable when S641 was mutated to an alanine (S641A). In order to validate these findings, we analyzed the binding of C-terminal HCN2 constructs (HCN2-CT and HCN2-CT-S641A) to TiO_2_ beads (Fig. 3D). TiO_2_ efficiently binds negatively charged peptides and hence, can be used to determine alterations of the ratio of (highly charged) phosphorylated versus (less charged) non-phosphorylated peptides. After precipitation by TiO_2_ and subsequent western blot analysis with anti-myc antibody, an intense 50 kDa band was detected in lysates containing myc-tagged HCN2-CT and cGKII (Fig. 3D, second lane). By contrast, only a weak band was obtained for HCN2-CT-S641A (Fig. 3D, third lane). Densitometric analysis revealed that the intensity of the HCN2-CT-S641A band was only 32.2±1.1% of that of the HCN2-CT band (n = 4, p<0.01). Weak bands were also obtained for HCN2-CT and HCN2-CT-S641A when cGKII was not present in the assay (Fig. 3D, fourth and fifth lane). Notably, the intensity of the bands obtained in the absence of cGKII did not differ between wild type and mutant C-termini. Taken together, these findings indicated that the strong interaction between HCN2-CT and the TiO_2_ beads was caused by cGKII-mediated phosphorylation of S641. The weak bands seen for HCN2-CT-S641A may reflect background phosphorylation by endogenous kinases. Alternatively, the HCN2 C-terminus may be acidic enough in its non-phosphorylated form to bind to some extent to the TiO_2_ beads.

### cGKII shifts the half maximal activation voltage of HCN2 to more negative values

**Figure 4:**
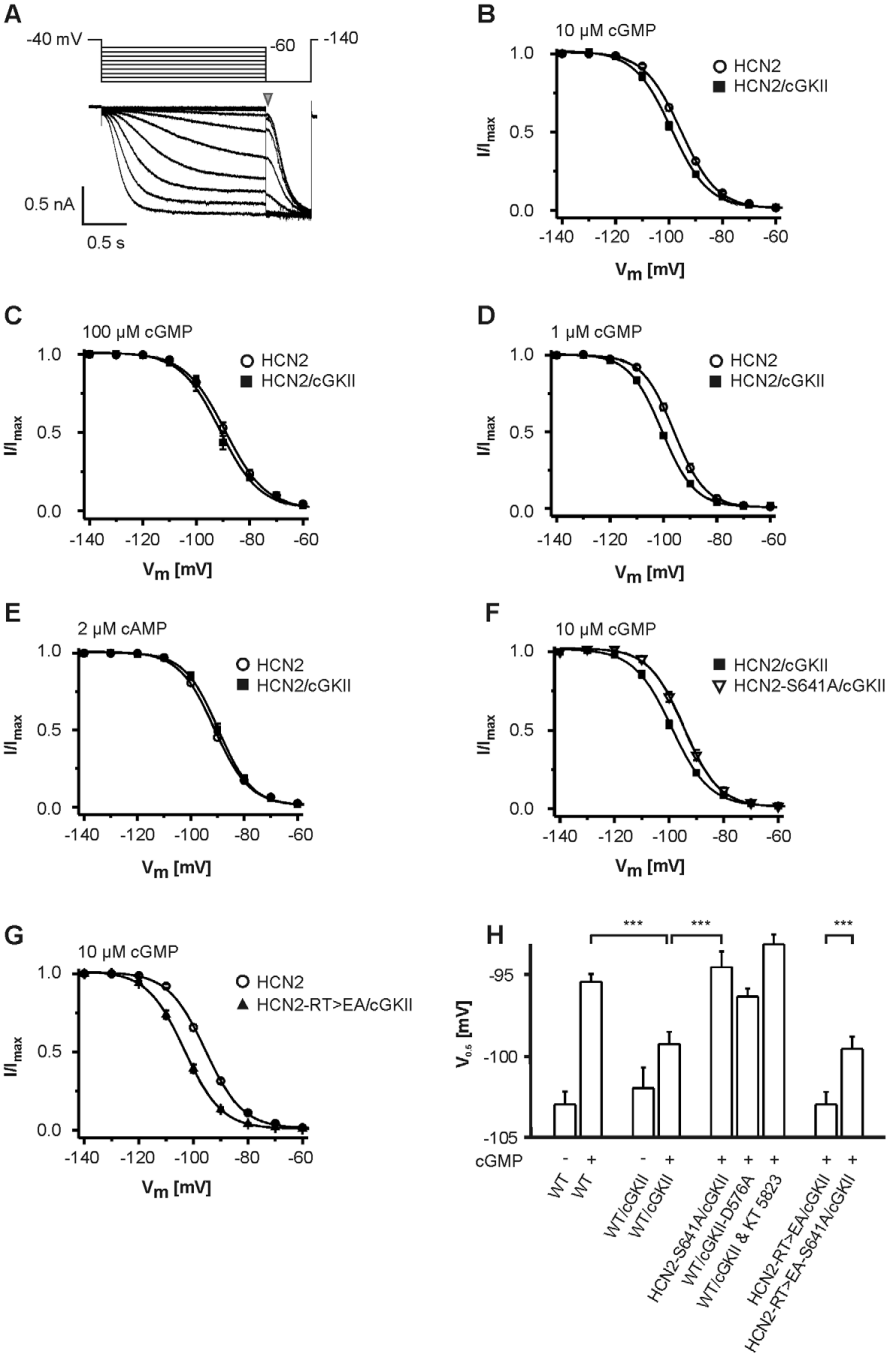
Regulation of voltage-dependence of HCN2 activation by cGKII. (**A**) Voltage step protocol and family of current traces of a HEK293 cell transiently transfected with HCN2. (**B–D**) Normalized current-voltage (IV) dependence of HCN2 activation in the presence and absence of cGKII. The voltage-dependence was determined in the presence of 10 µM intracellular cGMP (**B**), 100 µM intracellular cGMP (**C**) and 1 µM intracellular cGMP (**D**). (**E**) IV curves of HCN2 in the presence or absence of cGKII at 2 µM intracellular cAMP. (**F**) IV curves determined at 10 µM intracellular cGMP from cells coexpressing cGKII and HCN2 or HCN2-S641A. (**G**) IV curves of HCN2 compared to the IV curve of an HCN2 mutant with functionally impaired cyclic nucleotide binding domain (HCN2-RT>EA) that was coexpressed with cGKII. Currents were measured in the presence of 10 µM cGMP. (**H**) Comparison of midpoint potentials (V_0.5_) of wild type (WT) and HCN2 mutants (HCN2-S641A, HCN2-RT>EA). Channels were expressed alone or together with either wild type or catalytically inactive GKII (cGKII-D576A). V_0.5_ was determined from the normalized IV curves in the presence (+) or absence (−) of 10 µM cGMP as indicated. In one set of experiments the cGKII was inhibited by the pharmacological blocker KT5823. *** = p<0.001.

We next tested whether cGKII affects the properties of HCN2-mediated currents. To this end we transiently expressed wild type and mutant HCN2 in HEK293 cells and determined I_h_ by applying hyperpolarizing voltage steps from −140 mV to −60 mV in 10 mV increments from a holding potential of −40 mV for followed by a step to −140 mV (Fig. 4A). The presence of cGKII had no influence on current densities (at −140 mV: HCN2: −155±24.7 pA/pF, n = 15; HCN2/cGKII: −170±43.4 pA/pF, n = 7) nor did it influence the activation kinetics of HCN2-mediated currents (τ at −140 mV: HCN2: 293±15.2 ms, n = 15; HCN2/cGKII: 309±28.6 ms, n = 7). However, cGKII induced a hyperpolarizing shift of the voltage-dependence of activation of HCN2 currents in the presence of cGMP. At 10 µM cGMP which is close to the K_a_ (cGMP) of HCN2 [20] the shift was about −4 mV (V_0.5_ values at 10 µM cGMP. HCN2: −95.5±0.49 mV, n = 16; HCN2/cGKII: −99.3±0.74 mV, n = 13) (Fig. 4B). The hyperpolarizing shift was somewhat smaller at 100 µM cGMP (Fig. 4C) (ΔV = −2.2 mV; V_0.5_ values at 100 µM cGMP. HCN2: −89.2±1.02 mV, n = 8; HCN2/cGKII: −91.4±1.17 mV, n = 7) while it was more pronounced at a low cGMP concentration (Fig. 4D) (ΔV at 1 µM cGMP = −4.9 mV; V_0.5_ values at 1 µM cGMP. HCN2: −96.1±0.68 mV, n = 7; HCN2/cGKII: −101±0.35 mV, n = 7). No shift was observed at 2 µM cAMP (Fig. 4E). It is well known that the V_0.5_ value of HCN2 currents is shifted to more positive values by direct interaction of cGMP with the CNBD [2]. This direct cGMP-mediated voltage shift (ΔV_0.5_) was about +7.5 mV at 10 µM cGMP (Fig. 4H, first two columns). In the presence of cGKII the cGMP-mediated shift was reduced to about 4 mV (Fig. 4B, and Fig. 4H, third and fourth column) suggesting that cGKII counteracted the stimulatory effect exerted by direct binding of cGMP to the channel CNBD.

We next asked whether phosphorylation at S641 is required for the inhibitory effect of cGKII. In line with a crucial role of S641 for this inhibitory effect, the cGMP/cGKII-mediated hyperpolarizing shift of the V_0.5_ value was not observed in currents from HCN2-S641A channels (V_0.5_ value at 10 µM cGMP: −94.6±0.95 mV, n = 15) (Fig. 4F and Fig. 4H fourth and fifth column). Similarly, inactivation of cGKII by either introducing a point mutation in the catalytic domain (cGKII-D576A [21]) or by addition of a specific pharmacological blocker KT5823 abolished the inhibitory cGKII effect on WT HCN2 to a similar extent as the S641A mutation (Fig. 4H, sixth and seventh column). We finally asked whether the inhibitory effect of cGKII requires binding of cGMP to the CNBD or whether it is independent from the direct cGMP effect of the channel. To this end, we employed a HCN2 mutant (HCN2-RT>EA) that carries two amino acid replacements in the β7 strand of the CNBD (R591E and T592A) that are known to abolish cGMP binding and the cGMP-mediated shift of the V_0.5_ value of HCN2 currents to more positive values [22]. In the presence of 10 µM cGMP and the cGKII, the V_0.5_ of the HCN2-RT>EA mutant was much more negative than that of WT HCN2 (Fig. 4G, columns four and eight. V_0.5_ values at 10 µM cGMP. HCN2-RT>EA: −103±0.77 mV, n = 16). Importantly, however, introduction of the S641A mutation into the HCN2-RT>EA backbone again eliminated the hyperpolarizing shift by cGMP/cGKII and lead to a positive shift of the V_0.5_ compared to HCN2-RT>EA plus cGMP/cGKII (Fig. 4H, last two columns; ΔV_0.5_ = +3.4 mV; HCN2-RT>EA/cGKII: −103.0±0.77 mV, n = 16; HCN2-S641A-RT>EA/cGKII: −99.6±0.76 mV, n = 10).

## Discussion

**Figure 5:**
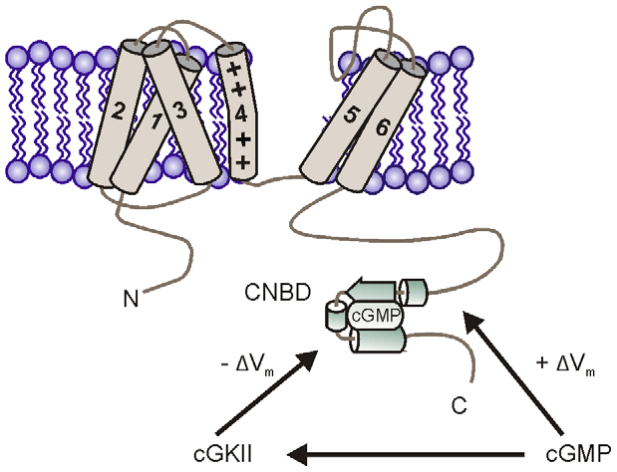
Model of the bidirectional regulation of HCN2 gating by cGMP. cGMP shifts the voltage-dependence of HCN2 activation to more positive voltage (+ΔV) via direct interaction with the CNBD of HCN2 and induces a hyperpolarizing shift (−ΔV) by activating cGKII that is bound to the channel.

So far, there are only a few reports on the regulation of I_h_ by cGMP. Pape et al. showed that NO/cGMP controls oscillatory activity in thalamocortical neurons via direct upregulation of I_h_ [23]. In addition, cGMP-mediated activation of sinoatrial I_h_ may induce an increase in heart rate [24]. In this study, we provide for the first time evidence for a bidirectional regulation of the HCN2 channel gating by cGMP (Fig. 5). It has long been known that cGMP, like cAMP, shifts the voltage-dependence of HCN channel activation to more positive values and, thereby, acts as a positive regulator of channel activity [2], [4]. Mechanistically, this regulation is conferred by direct binding of cGMP to the CNBD, which is allosterically coupled to the HCN channel activation gate. Our data indicate that cGMP can also act as a gating inhibitor via cGKII-dependent phosphorylation. We show that cGKII binds to the C-terminus of HCN2 and phosphorylates this channel at S641. Phosphorylation of S641 shifts the midpoint potential of HCN2 by about 4 mV to more hyperpolarizing values. The effect of cGKII is independent of the capability of the CNBD to bind cGMP since it also occurs in a HCN2 mutant with functionally impaired CNBD. In the absence of cGMP the V_0.5_ of WT and R591E/T592A channels are not statistically different from each other (for both V_0.5_ is ∼−103 mV). Introduction of S641A also does not change V_0.5_ with respect to WT in the absence of cGMP. Thus, one would not expect a negative shift exerted by the kinase per se. Indeed, our measurements demonstrate that in the absence of cGMP V_0.5_ of HCN2 is not altered by cGKII (see Fig. 4H, first and third column: V_0.5_ of HCN2 = −103 mV; V_0.5_ of HCN2+cGKII = −102 mV). By contrast, comparison of V_0.5_ of the HCN2-R591E/T592A channel with that of the HCN2-S641A/R591E/T592A triple mutant in the presence of cGMP (which is required to activate the kinase) clearly reveals the inhibitory effect of cGKII on HCN2 gating.

It is noteworthy to mention that the apparent cGMP affinity of HCN2 is about 30 times lower than that reported for cGKII (6 µM vs. 0.2 µM [20], [25]). Thus, at very low cGMP concentrations the inhibitory action of cGMP via cGKII may be dominant while at higher concentrations of cGMP the direct stimulatory effect more and more outweighs the inhibition imposed by the kinase. This hypothesis is supported by our finding that the hyperpolarizing shift induced by cGKII was more pronounced at low (1 µM) than at high (100 µM) cGMP concentration. Finally, our data indicate that low micromolar concentrations of cAMP that activate HCN2 currents by binding to the CNBD do not cross-stimulate cGKII and thus should not interfere with the cGKII-dependent modulation of HCN2.

The exact mechanism underlying the cGKII-mediated inhibition of channel gating remains to be determined. S641 is localized at the C-terminal end of the αC-helix of the CNBD, which has been shown to play an important role in HCN channel gating [26], [27]. One may speculate that the presence of the bulky negatively charged phosphate group could well interfere with the allosteric movement of the proximal C-terminus of HCN2 during channel gating. Other protein kinases, including Src kinase [16] and protein kinase A [15] have been also shown to regulate HCN channel gating via phosphorylation in the C-terminus. Thus, phosphorylation is probably a common cellular mechanism to modulate HCN channel activity.

We could provide direct experimental evidence for colocalization of HCN2 and cGKII in mouse brain. Given the widespread distribution of both proteins in brain [18], [19], [28], regulation of HCN2 by cGKII could be potentially relevant in many types of neurons. Given that S641 is highly conserved within the HCN channel family, this kind of regulation may be a commonality of HCN channels.

A modulatory action of cGKII on other ion channels was only established for the cystic fibrosis transmembrane conductance regulator (CFTR)-Cl^−^ channel and the AMPA receptor subunit GluR1. In both cases, cGKII was shown to increase the cell surface expression [29]. CFTR was also shown to be activated by cGMP/cGKII [30], [31]. Our coexpression studies in HEK293 cells and primary hippocampal neurons do not support an effect of cGKII on HCN2 trafficking and cell surface expression.

In conclusion, we provide evidence that the voltage-dependence of HCN2 activation is determined by complex interactions of multiple signaling pathways that control the concentration of cGMP and/or cAMP and the activity of cGKII. HCN2 is a key determinant of resting membrane potential in neurons and plays a key role in controlling neuronal excitability [8]. Thus, a cGKII-mediated phosphorylation and change of the value of half-maximal activation of HCN2 in neurons would immediately interfere with neuronal activity because it directly affects the threshold at which HCN2 can be activated.

It is tempting to speculate, that in addition to the well established up-regulation of I_h_ by cAMP and/or cGMP, neurons that express cGKII are also be able to down-regulate I_h_ via the NO/cGMP system by changing the ratio of phosphorylated versus non-phosphorylated HCN2 channels. This “dual modulation by cGMP” may have evolved to allow a tighter control of HCN channel activity, and thus, a better control of the threshold for activation of neurons.

## Materials and Methods

### Lentiviral and expression vectors

To construct the LV-Syn1.1-eGFP lentiviral vector plasmid, we replaced the CMV promoter sequence in LV-CMV-eGFP [32] with a rat synapsin 1 promoter sequence, which was PCR amplified from the FSy(1.1)GW plasmid (kindly provided by Pavel Osten, SUNY Downstate Brooklyn) [33]. LV-Syn1.1-HCN2 was prepared by replacing the eGFP sequence in LV-Syn1.1-eGFP with the mouse HCN2 cDNA [20]. The coding region of the mouse cGKII [34] was subcloned into the pcDNA3 (Invitrogen) and pIRES-EGFP (Clonetech), respectively. LV-Syn1.1-cGKII was prepared by replacing the eGFP sequence in LV-Syn1.1-eGFP with the C-terminally myc-tagged mouse cGKII cDNA. High titer replication-deficient lentiviral vector particles were generated as described previously [32]. Mutations in the HCN channel and cGKII were introduced by site directed mutagenesis (QuikChange II kit, Stratagene). An overview of the HCN2 channel constructs used in this study is shown schematically in Figures 1C and 3B.

### Mice

The HCN2-deficient mouse line was described previously [8] as well as the cGKII-deficient mouse line [35]. All animals have a mixed background of 129SvJ and C57-Bl6/N strains, received food and water ad libitum and lived in a light-dark cycle of 12 h. All mouse husbandry and experimental procedures were performed in accordance with the German animal protection standards and were approved by the Government of Upper Bavaria (Regierung von Oberbayern, Munich, Germany) and the permit number is 55.2-1-54-2531-88-05.

### Cell culture

HEK293 and COS7 (DSMZ) cells were maintained in Dulbecco's modified Eagle's medium (Invitrogen) supplemented with 10% fetal bovine serum (Biochrom AG), 100 units/ml penicillin and 100 units/ml streptomycin (Biochrom AG) at 37°C and 10% CO_2_. For biochemical experiments, HEK293 or COS-7 cells were transfected using the calcium phosphate method and for electrophysiological experiments the FuGene6 transfection reagent (Roche) was used.

### Primary hippocampal neuron culture

Primary neurons of C57-Bl6/N mice were isolated as described previously [36]. Briefly, brains of mouse embryos were dissected on embryonic day 16.5 (E16.5) and the hippocampi were isolated. After incubation with trypsin-EDTA, the tissue was dissociated. Approximately 100,000 primary hippocampal neurons were plated on acid-washed, poly-l-lysine treated coverslips. The culture was maintained at 36.5°C and 5% CO_2_ in N2-medium (N-MEM containing N2 supplement (Invitrogen) and 200 g/l ovalbumin (Sigma)). On day two after plating, the primary neuron culture was transduced with lentiviral particles expressing either a cGKII-myc fusion protein or HCN2.

### Immunocytochemistry

Except slight modifications, staining of primary hippocampal neurons was performed as described previously [37]. Cells were fixed with 4% paraformaldehyde containing 4% sucrose in phosphate buffered saline (PBS) at room temperature for 20 min. Subsequently, cells were quenched with 50 mM ammonium chloride, permeabilized with 0.1% Triton X-100 (Roth) and blocked with 2% fetal bovine serum and 2% bovine serum albumin containing 0.2% cold water fish gelatin. Primary neurons were incubated over night at 4°C with anti-HCN2 (Alomone) and anti-myc (clone 9B11, Cell Signaling) diluted in 5% Chemiblocker (Chemicon). After excessive washing with PBS, the cells were incubated for 1 h at room temperature with secondary antibodies, Cy3αrb and Cy2αms (Jackson) diluted in 2% Chemiblocker. Hoechst 33342 DNA stain (Invitrogen, 5 µg/ml) was used to visualize cell nuclei. The cells were examined on a confocal laser scanning microscope (LSM510 Meta, Zeiss).

### Immunohistochemistry

Twelve µm thick coronal cryosections of adult mouse brain were fixed with 4% paraformaldehyde in PBS and blocked with PBS containing 10% normal goat serum (Vector laboratories) and 0.3% Triton X-100. Anti-HCN2 (Alomone) and anti-cGKII [38] were used as primary antibodies. Endogenous peroxidase activity was quenched (3% H_2_O_2_ in methanol) before sections were incubated with horseradish peroxidase-conjugated anti-rabbit IgG antibodies (Jackson). Tyramide signal amplification was performed according to manufacturer's instruction using Cy3-conjucated tyramide (TSA-Plus Cyanine 3 System, Perkin Elmer). To visualize cell nuclei, slices were counter stained with 5 µg/ml Hoechst 33342. The brain slices were examined on an epifluorescence microscope (Axioplan 2, Zeiss) and a confocal laser scanning microscope (Leica TCS LSI).

### Coimmunoprecipitation

For protein analysis, transfected HEK293 cells or dissected brains were homogenised in lysis buffer (50 mM Tris-HCl pH 7.4, 150 mM NaCl, 1 mM EDTA, 1% Triton X-100) containing proteinase inhibitors (PI) (complete, EDTA-free proteinase inhibitor cocktail tablets, Roche) and centrifuged (13,000 rpm, 4°C, 15 min) to remove cell debris. The amount of protein in the supernatant was determined using the Bradford assay. Brain membrane fractions or cell lysates of HEK293 cells were incubated overnight at 4°C with protein A-Sepharose beads (Sigma) and a specific antibody. Beads were pelleted by centrifugation and washed three times with cold buffer (20 mM Tris, 5 mM MgCl_2_, 0.2 mM EDTA, 20% glycerine, 300 mM KCl; pH 7.9). The proteins were boiled for 5 min in Laemmli sample buffer, separated by SDS-PAGE and transferred to PVDF (Whatman). The western blot analysis was performed with rabbit anti-HCN2 L [8], rabbit anti-cGKII (kindly provided by Peter Ruth, Tübingen, Germany) or mouse anti-myc (clone 9E10, Cell Signaling Technologies).

### GFP-Trap assay

HEK293 cells were co-transfected with one of the C-terminal HCN2 myc-tagged constructs together with the C-terminally GFP-tagged cGKII construct (cGKII-GFP-pcDNA3). This combination gave the most robust results in co-immunoprecipitation experiments with C-terminal fragments of HCN2. While the anti-cGKII antibody nicely pulled down complexes of full length HCN2 and cGKII, it was less efficient in precipitating complexes between cGKII and C-terminal fragments of HCN2. Cell lysis was performed as described above. GFP-Trap-A (Chromotek) was performed according to manufacturer's instruction.

### TiO_2_ pulldown

TiO_2_ Mag Sepharose beads (GE healthcare) were used according to manufacturer's instructions with adaptations. 500 mg cell lysates from HEK293 cells expressing cGKII and/or myc-fusion proteins of wild type HCN2 (HCN2-CT) or HCN2-S641A (HCN2-CT-S641A) C-terminus were incubated for 30 min in binding buffer (1 M glycolic acid, 10 mM HEPES, pH 7.35, 3 µM 8-pCPT-cGMP (Calbiochem) and phosphatase inhibitors (PhosSTOP, Roche). Subsequently, the beads were precipitated using a magnetic rack and washed in 10 mM HEPES (pH 7.35) than boiled for 5 min in Laemmli sample buffer, and proteins were separated by SDS PAGE. The PVDF membrane was probed with a mouse anti-myc antibody (clone 9E10, Cell Signaling Technologies).

### Kinase assay

The procedure of the *in vitro* kinase assay was described previously [39]. In COS-7 cells the cGKII is a soluble protein in contrast to the membrane-bound cGKII in HEK293 cells [34], [40]. For the kinase assay, COS-7 cells were transiently transfected using the calcium phosphate method. The HCN2 wild-type protein was compared to the HCN2 mutants HCN2-S641A and HCN2-756STOP in the presence or absence of cGKII. To obtain cell lysates, cells were washed twice with PBS and harvested. The cells were suspended in hypoosmotic lysis buffer (10 mM K_3_PO_4_, pH 7.4). Complete cell destruction was achieved by three times passing through a 27-gauge syringe needle and an additional freeze–thaw cycle. Lysates containing 30 µg of protein were incubated in 50 mM 2-(N-morpholino)ethanesulfonnic acid (MES) pH 6.9, 10 mM NaCl, 1 mM Mg^2+^ acetate, 0.4 mM EGTA, 0.1% Triton X-100 and 3 µM 8-pCPT-cGMP (Calbiochem). The reaction was started by adding 0.1 mM [γ-^32^P]-ATP (2,000 cpm/pmol, PerkinElmer). After incubation for 15 s up to 10 min at 30°C, the reaction was stopped by addition of Laemmli buffer and denaturation at 95°C for 5 min. Proteins were separated by SDS-PAGE and blotted onto a PVDF membrane. Incorporated radioactivity was visualized by autoradiography and phosphoimage analysis (BASReader 2.9, Raytest).

### Electrophysiology

Currents of heterologously expressed HCN channels were measured in HEK293 cells at room temperature 2–3 days after transfection using the whole cell voltage-clamp technique. The extracellular solution was composed of (in mM): 110 NaCl, 0.5 MgCl_2_, 1.8 CaCl_2_, 5 HEPES, 30 KCl, pH 7.4 adjusted with NaOH. The intracellular solution contained (in mM): 130 KCl, 10 NaCl, 0.5 MgCl_2_, 1 EGTA, 5 HEPES, 3 Mg-ATP, 0.5 Na-GTP, pH 7.4 adjusted with KOH. Pipettes were pulled from borosilicate glass capillaries (GC150TF, Harvard Apparatus) and had resistances of 2–3 MΩ when filled with the intracellular solution. The cGMP-dependent kinase inhibitor KT5823 was purchased from Cayman chemicals. A 1 mM stock solution was prepared in ethyl acetate and was freshly diluted to a final concentration of 1 µM in extracellular solution before use.

Data were acquired at 10 kHz using an Axopatch 200B amplifier and pClamp10.2 (Molecular Devises). Voltage clamp data were analyzed off-line by using Clampfit 10.2 (Molecular Devises). Steady-state activation curves were determined by hyperpolarizing voltage steps from −140 mV to −60 mV in 10 mV increments from a holding potential of −40 mV for 1.8 s followed by a step to −140 mV (Fig. 4A). Currents measured immediately after the final step to −140 mV, were normalized by the maximal current (I_max_) and plotted as a function of the preceding membrane potential (Fig. 4A, indicated by a grey arrow). The data points were fitted with the Boltzmann function: (I−I_min_)/(I_max_−I_min_) = {1−exp[(V_m_−V_0.5_)/*k*]} where I_min_ is an offset caused by a nonzero holding current, V_m_ is the test potential, V_0.5_ is the membrane potential for half-maximal activation, and *k* is the slope factor.

### Statistics

All biochemical experiments were repeated at least three times with protein samples obtained from three independent transfections or tissue preparations. For the quantification of the results obtained in independent TiO_2_ pulldown assays, the intensity of western blot bands was determined by counting the pixels per area for HCN2-CT and HCN2-CT-S641A with or without cGKII, respectively. The values were averaged and normalized to HCN2-CT with or without cGKII, respectively. For electrophysiological data, the statistical analysis was performed by one-way ANOVA. Data are presented as mean ± S.E.M. (*n* = number of recorded cells). Values of p<0.01 were considered significant.
